# Systems chemo-biology analysis of DNA damage response and cell cycle effects induced by coal exposure

**DOI:** 10.1590/1678-4685-GMB-2019-0134

**Published:** 2020-06-26

**Authors:** Jose F. Torres-Ávila, Lyda Espitia-Pérez, Diego Bonatto, Fernanda Rabaioli da Silva, Iuri Marques de Oliveira, Luís F.O. Silva, Dione Silva Corrêa, Johnny Ferraz Dias, Juliana da Silva, João Antonio Pêgas Henriques

**Affiliations:** 1 Universidade Federal do Rio Grande do Sul, Centro de Biotecnologia, Departamento de Biofísica, Porto Alegre, RS, Brazil; 2 Universidad Simón Bolívar, Facultad de Ciencias Básicas y Biomédicas, Barranquilla, Colombia; 3 Universidad del Sinú, Grupo de Investigación Biomédica y Biología Molecular, Montería, Córdoba, Colombia; 4 Centro de Biotecnologia da Universidade Federal do Rio Grande do Sul, Departamento de Biologia Molecular e Biotecnologia, Porto Alegre, RS, Brazil; 5 Universidade La Salle, Canoas, RS, Brazil; 6 Universidad de la Costa, Civil and Environmental Department, Barranquilla, Colombia; 7 Universidade Luterana do Brasil, Programa de Pós-Graduação em Genética e Toxicologia Aplicada, Centro de Pesquisa de Produtos e Desenvolvimento, Canoas, RS, Brazil; 8 Universidade Federal do Rio Grande do Sul, Instituto de Física, Laboratório de Implantação de Íons, Porto Alegre, RS, Brazil; 9 Universidade Luterana do Brasil, Laboratório de Toxicologia Genética, Canoas, RS, Brazil; 10 Universidade de Caxias do Sul, Instituto de Biotecnologia, Laboratório de Genômica, Proteômica e Reparo de DNA, RS, Brazil

**Keywords:** Coal, Colombia, cell cycle, systems chemo-biology

## Abstract

Cell cycle alterations are among the principle hallmarks of cancer. Consequently, the study of cell cycle regulators has emerged as an important topic in cancer research, particularly in relation to environmental exposure. Particulate matter and coal dust around coal mines have the potential to induce cell cycle alterations. Therefore, in the present study, we performed chemical analyses to identify the main compounds present in two mineral coal samples from Colombian mines and performed systems chemo-biology analysis to elucidate the interactions between these chemical compounds and proteins associated with the cell cycle. Our results highlight the role of oxidative stress generated by the exposure to the residues of coal extraction, such as major inorganic oxides (MIOs), inorganic elements (IEs) and polycyclic aromatic hydrocarbons (PAH) on DNA damage and alterations in the progression of the cell cycle (blockage and/or delay), as well as structural dysfunction in several proteins. In particular, IEs such as Cr, Ni, and S and PAHs such as benzo[a]pyrene may have influential roles in the regulation of the cell cycle through DNA damage and oxidative stress. In this process, cyclins, cyclin-dependent kinases, zinc finger proteins such as TP53, and protein kinases may play a central role.

## Introduction

One of the largest open-pit coal mines in the world is located in northern Colombia Huertas *et al.* (2012a). According to the 2015 BP Statistical Energy Survey, Colombia aimed to increase its coal production by 35% to 115,000 tons per year by 2015 from 85,000 tons in 2011. Open-pit mines were forcast to account for almost 50% of this increase (BP, 2014). According to Chaulya (2004) and Huertas *et al.* (2012b), activities associated with coal extraction during surface coal mining release major air pollutants into the atmosphere as particulate matter (PM) and coal dust. These activities include: topsoil removal, drilling, blasting, overburden loading and unloading, coal transport over unpaved roads and wind erosion of exposed surfaces. In addition to the coal itself, PM and coal dust around coal mines can also contain O, N, H, trace species, and several inorganic minerals. The trace species may include SiO_2_, Cu, Al, Ni, Cd, B, Sb, Fe, Pb, and Zn (Huertas *et al.*, 2012a). In mining, excessive occupational exposure to metals is considered to be the leading cause of metal-related cancers (Gloscow, 2007). Additionally, in open-cast coal mines, coal is stored at elevated ambient temperatures, where combustion may lead to the emission of polycyclic aromatic hydrocarbons (PAHs) (Liu *et al.*, 2008), most of which exhibit mutagenic and carcinogenic properties (Celik *et al.*, 2007).

There is a growing body of evidence that links long-term exposure to coal mining residues with increased risks of cardiovascular mortality (Pope and Dockery, 2006; Brook *et al.*, 2010), premature mortality (Callén *et al.*, 2009) and cancer (Pope 3rd *et al.*, 2002, 2011). However, the mechanisms underlying the development of these adverse effects are poorly understood. In vitro toxicological studies have found that exposure to PM induces cell damage including genotoxicity (de Kok *et al.*, 2005; Billet *et al.*, 2008), cell death (Hsiao *et al.*, 2000; Alfaro-Moreno *et al.*, 2002), cell cycle alterations (Poma *et al.*, 2006) and the stimulation of pro-inflammatory cytokine production (Schins and Borm, 1999). Some of the mechanisms proposed for these effects include the occurrence of oxidative damage through the production of reactive oxygen species (ROS) (Valko *et al.*, 2006); the release of growth factors, such as TGF-β (Borm, 1997; Sambandam *et al.*, 2015), and reduced proliferation associated with cell cycle arrest in response to genotoxic stresses and structural dysfunction of proteins (Kocbach *et al.*, 2008; Gualtieri *et al.*, 2011). Furthermore, a recent study (Espitia-Pérez *et al.*, 2018) revealed a highly significant correlation between PM_2.5_ levels around the coal mining areas of northern Colombia and incidences of mitotic arrest, centromere damage, kinetochore malfunction and disruption of the mitotic spindle in local populations.

It has been shown that oxidative stress can override the spindle checkpoint (D’Angiolella *et al.*, 2007), inducing microtubule depolymerization (Parker *et al.*, 2014) and alterations in the spindle structure (Choi *et al.*, 2007). This observation supports prior results showing that the organic components of PM_2.5_, particularly PAHs, have deleterious effects on the cell cycle and cause DNA damage (Longhin *et al.*, 2013). DNA-integrity checkpoints G1/S and G2/M and metaphase–anaphase (M/A) transitions are particularly implicated in cell cycle delay (Branzei and Foiani, 2008).

Considering that one of the main characteristics of cancer is cell cycle alterations (Otto and Sicinski, 2017). The study of cell cycle regulators, particularly in terms of exposure to environmental stresors, has emerged as a pertinent avenue of research in cancer studies (Puente *et al.*, 2014). Populations are rarely exposed to single air pollutants; therefore, experimental investigations which have focused on single-pollutant effects do not accurately assess real-world exposure risks. Consequently, a multi-pollutant perspective should be the focus of air quality management, rather than adhering to a single-pollutant viewpoint (Huang *et al.*, 2012). Furthermore, although several recent studies have investigated the combined toxicity of complex mixtures of chemicals (Labranche *et al.*, 2012), detailed investigations into synergistic toxicity and the possible mechanisms involved in biological responses to complex exposures remain scarce (Ku *et al.*, 2017). Therefore, in the present study, we performed a chemical analysis of mineral coals from two different Colombian mines to identify the main compounds present. We then performed systems chemo-biology analyses to reveal the interactions between these compounds and proteins associated with the cell cycle, elucidating their underlying regulatory mechanisms.

## Material and Methods

### Coal sample collection

To construct a chemo-biology interactome network for the proteins associated with the cell cycle and the major chemical constituents present in the coal samples, we chemically characterized bituminous and sub-bituminous coal samples, each collected from a different open-pit mine in Colombia. The samples were collected from coalfaces at the ‘El Cerrejón’ (La Guajira, Colombia) and ‘Guacamaya’ (Puerto Libertador, Córdoba, Colombia) coal mines in December 2013 (Figure S1). Six random points at each mine were sampled; samples were then prepared as a homogeneous pool. Coals from El Cerrejón are typically bituminous with a volatile content of 37.4% and an ash content of 6.8% (dry basis) (Feng *et al.*, 2003). Coals from Guacamaya are sub-bituminous with a high S content (2.30% total S with 1.06% as pyritic, 1.10% as organic and 0.14% from sulfates) and high volatile content (Prada *et al.*, 2016). While detailed chemical characterizations of El Cerrejón coal have been reported elsewhere (Nathan *et al.*, 1999), other Colombian coals, such as those obtained from the Guacamaya mine, have not been sufficiently characterized.

### Analytical methods

Chemical analysis of the coal samples included identification of the major inorganic oxides (MIOs) in the coal ashes, inorganic element (IE) determination and quantification of PAHs, described in detail below.

### Analysis of MIOs in coal ashes

A fraction of bituminous and sub-bituminous coal samples were incinerated separately at 815°C. The resulting ashes were processed according to the methods described by Norrish and Hutton (1969). Finally, the detection of MIOs was performed using X-ray fluorescence spectrometry (XRF) in a Philips PW2400 spectrometer system equipped with SuperQ software.

### IE measurements by particle-induced X-ray emission (PIXE) assay

The elemental composition of each coal sample was measured by the conventional in vacuo PIXE assay, as described by Johansson *et al.* (1995). Individual portions of each coal sample were homogenized using a mortar, pressed into pellets, and then placed in the reaction chamber (at ∼ 10-5 mbar), in a 3-MV Tandetron accelerator equipped with an energy resolution of ∼ 155 eV to 5.9 keV for obtaining the spectra. The spectra were analyzed using GUPIXWIN software (Campbell *et al.*, 2010), and expressed in parts per million. Each sample was evaluated three times in independent replicates to obtain the mean and standard deviation.

### Measurement and quantification of PAHs

The PAH contents of the coal samples were quantified using the HPLC-UV/Vis method, according to Sun *et al.* (1998) and Cavalcante *et al.* (2008). Briefly, 5 g of each coal sample was dried at 30 °C for 24 h (in duplicate) for later extraction. The extraction was performed by ultrasonication in 5 mL acetone/hexane (1:1, *v/v*) for 15 min. The filtrate was concentrated on a rotary evaporator and then further under a stream of nitrogen gas to ∼2 mL. A clean glass column was used for adsorption chromatography. The concentrated extracts were fractionated using a 20 × 1.5-cm column containing pre-cleaned silica gel (20 h at 110 °C). The column was first eluted with 20 mL hexane/dichloromethane (9:1, *v/v*), then with 30 mL hexane/dichloromethane (4:1, *v/v*) and finally with 10 mL dichloromethane/methanol (9:1, *v/v*). The eluted volumes were reduced to 1 mL, and finally, each extract was injected into a HPLC-UV system. The chromatographic conditions were as follows: 5 μm Kromasil C18 reverse-phase column (250 × 4.6 mm); injection volume: 20 μL; mobile phase (A): acetonitrile; mobile phase (B): MilliQ water; gradient method: 0 min (1:1), 10 min (7:3), 20 min (8:2), 25 min (8:2), 28 min (1:1), 30 min (1:1) and λ = 254 nm. Analytical curves were created using external standardization for quantification. In our study, we detected 11 PAHs in the samples. The PAHs detected and their limits of detection were: naphthalene (1.7976 g L^−1^), acenaphthylene (0.0041 g L^−1^), phenanthrene (0.1758 g L^−1^), anthracene (0.0339 g L^−1^), fluoranthene (0.3787 g L^−1^), benzo[a]anthracene (0.3411 g L^−1^), benzo[b]fluoranthene (0.0691 g L^−1^), dibenzo[a,h]anthracene (1.1110 g L^−1^), benzo[k]fluoranthene (2.2221 g L^−1^), indene[1,2,3-cd]pyrene (3.5788 g L^−1^) and benzo[g,h,i]perylene (0.0005 g L^−1^). All chromatographic measurements were performed in duplicate at ambient temperature.

### Interactome data mining and design of the chemo-biology network

To design the interactome network among the main chemical substances present in the coal samples and their potential interactions with *Homo sapiens* proteins involved in the cell cycle, we used the STITCH search engine version 5.0 [http://stitch.embl.de/] and STRING 10.0 [http://http://string-db.org/newstring_cgi/show_input_page.pl/] (Snel *et al.*, 2000; Jensen *et al.*, 2008). A total of 36 chemical elements were detected in the chemical analysis of both coal samples using the XRF, PIXE, and HPLC/UV/Vis methods, and these were used for the exploration of networks within the STITCH metasearch engine. While STITCH allows visualization of the physical interactions between chemical elements and proteins, the STRING metasearch engine generates protein-protein interactions (PPIs) (Feltes *et al.*, 2013). Each chemical–protein interaction (CPI) and PPI has a confidence level between 0 and 1.0 (where 1.0 indicates the highest confidence). Parameters used by the STITCH and STRING metasearch engines were as follows: all predictive methods were enabled except text mining; interactions: 50; degree of confidence: 0.7 and network depth: 1. The results were combined and analyzed using Cytoscape 3.4.0 (Shannon *et al.*, 2003) and the search engine GeneCards (Rebhan *et al.*, 1997; Safran *et al.*, 2010) using the default parameters.

The chemical elements not involved in interactions according to STITCH were excluded. Then, using Cytoscape 3.4.0., we created the interactome that fused the small CPI and PPI networks (not shown individually) that were generated by STITCH and STRING, respectively.

### Centrality analysis

To evaluate the node degree, betweenness, and to identify the ‘central’ nodes (chemical compounds/proteins) in the interactome, a centrality analysis of the interactome was performed using CentiScaPe 2.1 in Cytoscape (Scardoni *et al.*, 2009).

### Modular analysis of the major CPI-PPI network

In the interactome or CPI-PPI network, we analyzed clusters or highly connected regions that are indicative of functional protein complexes. These regions were identified using the Molecular Complex Detection application (MCODE) (Bader and Hogue, 2003; Scott, 2017). The MCODE application is included within the Cytoscape program and was used with the following parameters: loops; grade limit: 2; cluster expansion by a neighbor shell allowed; removal of a single connected node from the clusters; cut-off node density: 0.1; node score limit: 0.2; score: 2 and maximum network depth: 100.

### Gene ontology (GO) analysis

The genetic ontology analysis was performed using the Biological Networks GO tool (BiNGO 3.0.3) (Maere *et al.*, 2005), which is an application installed in Cytoscape. The clusters obtained with MCODE were analyzed to determine the main bioprocesses associated with each cluster. The degree of functional enrichment was evaluated quantitatively using the hypergeometric distribution by group and category (p-value). The false discovery rate algorithm (Benjamini and Hochberg, 1995) was used to correct for multiple tests, as implemented in BINGO with a significance of p <0.05.

### Comet assay

The alkaline comet assay was carried out according to Singh *et al.* (1988) and Tice *et al.* (2000) with several modifications for a high-throughput comet assay version, which allows the processing of multiple samples (Tice *et al.*, 2000). The high-throughput “96-mini gel format” is an 8x12 multi-array on GelBond® film (Lonza, Rockland Inc. ME, USA) (McNamee, 2000) described by Kiskinis *et al.* (2002). Briefly, 6 × 10^4^ V79 cells per well were seeded in 12-well cell culture plates and incubated for 24 h; plates were subsequently treated with a 0.15 mg/mL coal dilution from either El Cerrejón or Guacamaya for 24 h. The negative control was incubated with DMEM medium (FBS free), and the positive control was treated with 150 μM H_2_O_2_ for 3 h. For semi-automated scoring, stained cells were analyzed using an Olympus BX51 fluorescence microscope (Olympus, Japan) and examined at 40X magnification under a green filter (540 nm). We analyzed 100 randomly selected nuclei, 50 from each of the two replicate slides (Gutzkow *et al.*, 2013). % tail DNA was scored using the Comet Assay IV software (Perceptive Instruments, Haverhill, UK). The alkaline comet assay using the lesion-specific enzyme Formamidopyrimidine DNA glycosylase (FPG) (New England Biolabs, MA, USA) was used to detect oxidized purines (Collins, 2009). The protocol was used as previously described with minor modifications for the high-throughput comet assay (Kushwaha, 2011). FPG recognizes oxidized purines, specifically 8-oxo-guanine (Kushwaha, 2011). All experiments were performed in triplicate.

The normality of the data was evaluated using the Kolmogorov–Smirnov test, while the Student's *t*-test was used to compare results of the comet assay with and without the FPG enzyme. *P* ≤ 0.05 was considered statistically significant. All analyses were performed using the Graphpad PRISM statistical software (Graphpad Inc., San Diego, CA).

## Results

### Chemical characterization, interactome data mining and design of the chemo-biology network

The chemical characterizations of the El Cerrejón and Guacamaya coal samples are shown in Tables S1–S3. Chemical analysis by XRF revealed a similar oxide composition for each coal ash (Table S1). A total of 10 different oxides were identified. As expected, samples from El Cerrejón showed a bulk chemical composition containing several metal oxides in the order SiO_2_ > Al_2_O_3_ > Fe_2_O_3_ > K_2_O > MgO. Ashes from the sub-bituminous coal samples from Guacamaya showed higher concentrations of CaO, MgO, and SO_3_ and lower concentrations of SiO_2_ and Al_2_O_3_ than those reported in similar studies on bituminous and sub-bituminous coals (Blissett and Rowson, 2012).

As shown in Table S2, 15 IEs were identified by PIXE. Typically, bituminous samples from El Cerrejón showed higher concentrations of Si, Al, S, and Fe than those from the sub-bituminous samples of Guacamaya. Conversely, relatively high concentrations of Na, Ca, and Mg were present in the Guacamaya samples. Sr was detected only in the Guacamaya samples. Finally, concentration data for the 11 PAHs identified by HPLC/UV/Vis are shown in Table S3. For both samples, the most abundant PAHs detected were naphthalene, phenanthrene, anthracene, fluoranthene and benzo[a]anthracene. In general, however, higher concentrations of all PAHs were found in the El Cerrejón samples.

**Figure 1:**
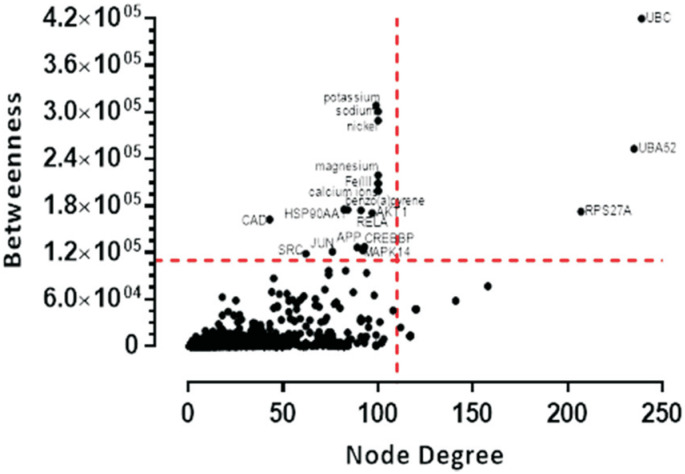
Scatter plot of degree and betweenness values for all nodes. Hubs (high degree), bottlenecks (high betweenness), and nodes with high relative values in both parameters are identified.

Chemical characterization of the bituminous and sub-bituminous coal samples revealed no significant differences in their chemical compositions. 36 compounds (i.e., 10 MIOs detected in coal ash, 15 IEs, and 11 PAHs) were used to construct the chemo-biology interactome. Once unconnected compounds were excluded, the remaining 24 protein-interacting compounds were used to generate 48 small CPI-PPI networks using the STRING and STITCH metasearch engines (Table 1). All the small networks were combined, resulting in a large CPI-PPI network with 2,057 nodes and 24,957 edges (Figure S2). This large CPI-PPI network was then analyzed using CentiScaPe 2.1 to identify the nodes (proteins) occupying central positions in the network architecture. In this context, nodes known as hub-bottlenecks (HBs) are the most important and combine hub (high degree) and bottleneck (high betweenness) characteristics according to Azevedo and Moreira-Filho (2015). Through centrality analysis, we observed three HB nodes (UBC, UBA52, and RPS27A) and 15 bottlenecks (HSP90AA1, CAD, SRC, JUN, MAPK14, APP, CREBBP, AKT1, K, Na, Ni, Mg, Fe, benzo[a]pyrene and Cr) (Figure 1 and Table S4).

**Table 1:** Chemical constituents of coal samples found in the major CPI-PPI network.

Compound	Classification	Chemical classification
Acenaphthene	Organic	Polycyclic Aromatic Hydrocarbon
Anthracene	Organic	Polycyclic Aromatic Hydrocarbon
Benzo(a)pyrene	Organic	Polycyclic Aromatic Hydrocarbon
Benzo(b)fluoranthene	Organic	Polycyclic Aromatic Hydrocarbon
Fluoranthene	Organic	Polycyclic Aromatic Hydrocarbon
Naphthalene	Organic	Polycyclic Aromatic Hydrocarbon
Phenanthrene	Organic	Polycyclic Aromatic Hydrocarbon
SiO_2_	Inorganic	Oxide of silicon
TiO_2_	Inorganic	Oxide of titanium
Fe_2_O_3_	Inorganic	Oxide of iron
Al	Inorganic	Metal
Ca	Inorganic	Alkaline earth metal
Cl	Inorganic	Halogen
Cr	Inorganic	Transition metal
Fe	Inorganic	Transition metal
K	Inorganic	Alkali metal
Mg	Inorganic	Alkaline earth metal
Mn	Inorganic	Transition metal
Na	Inorganic	Alkali metal
Ni	Inorganic	Transition metal
S	Inorganic	Non-metal
Sr	Inorganic	Alkaline earth metal
Ti	Inorganic	Transition metal
Zn	Inorganic	Transition metal

**Figure 2:**
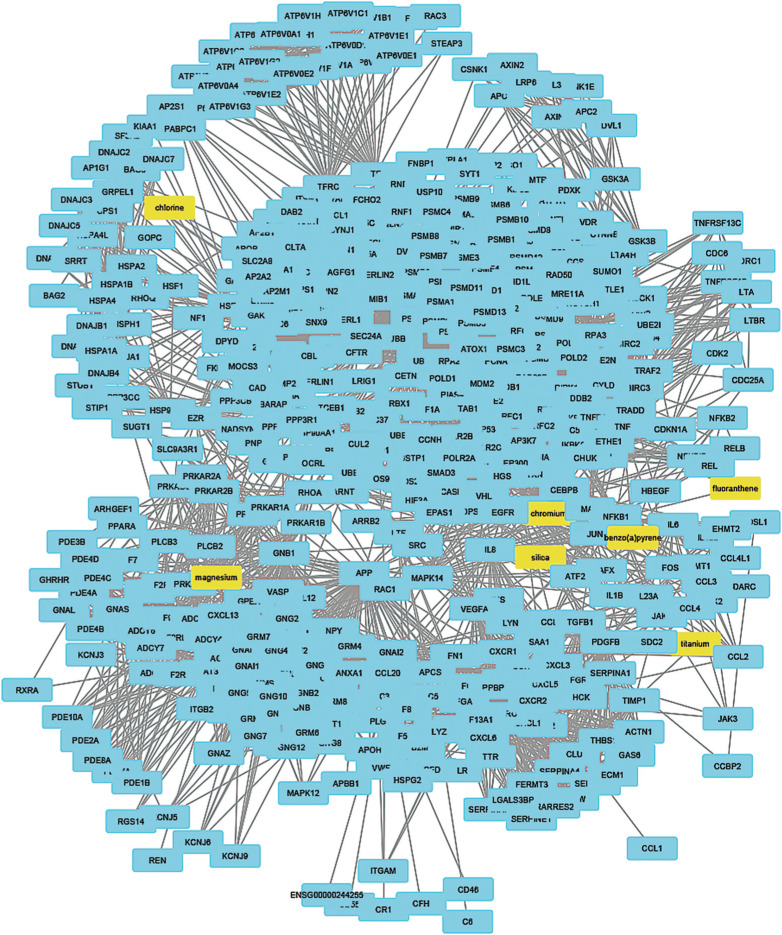
Cluster analysis of the major CPI-PPI network showing the association of cluster 6 with MIOs, IEs, and PAHs (yellow). The cluster is composed of 487 nodes and 5,545 edges, with Ci = 22,725. The associated constituents are SiO_2_, Ti, Mg, Cr, Cl, fluoranthene, and benzo[a]pyrene.

**Figure 3:**
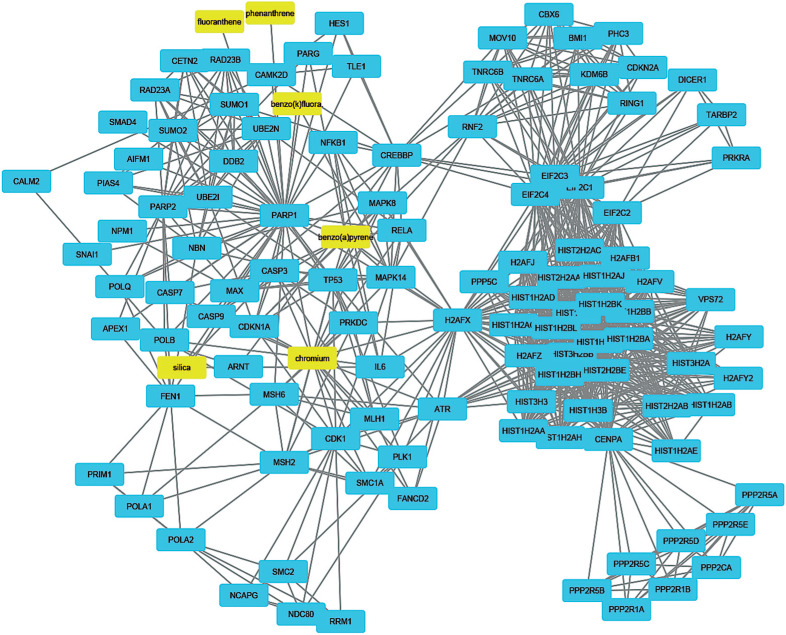
Cluster analysis of the major CPI-PPI network showing the association of cluster 11 with MIOs, IEs, and PAHs (yellow). It is composed of 117 nodes and 867 edges, with Ci = 14,695. The associated constituents are SiO_2_, Cr, benzo[b]fluoranthene, fluoranthene, phenanthrene and benzo[a]pyrene.

**Figure 4:**
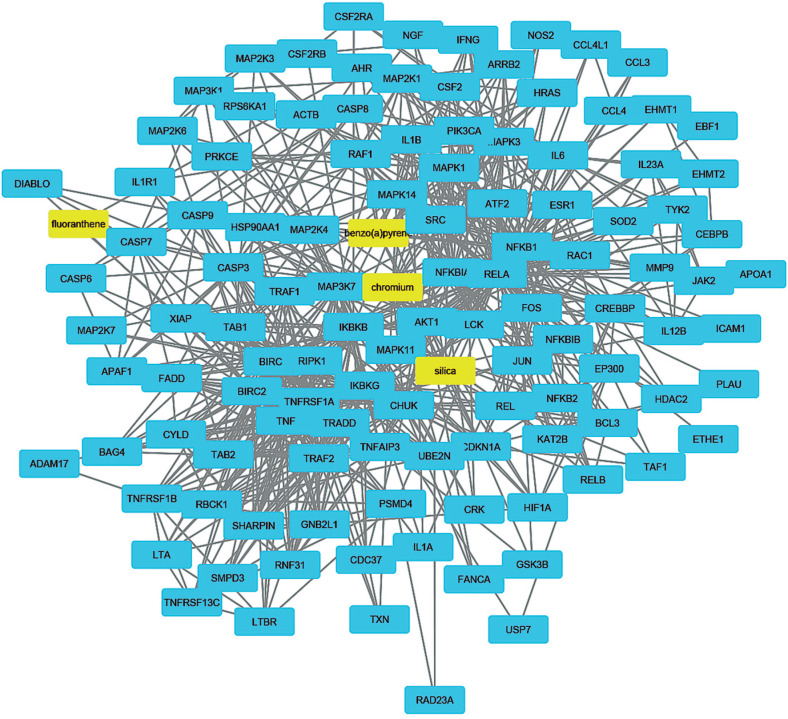
Cluster analysis of the major CPI-PPI network showing the association of cluster 13 with MIOs, IEs, and PAHs (yellow). It is composed of 118 nodes and 732 edges, with Ci = 12,303. The associated constituents include SiO_2_, Cr, fluoranthene, and benzo[a]pyrene.

**Figure 5:**
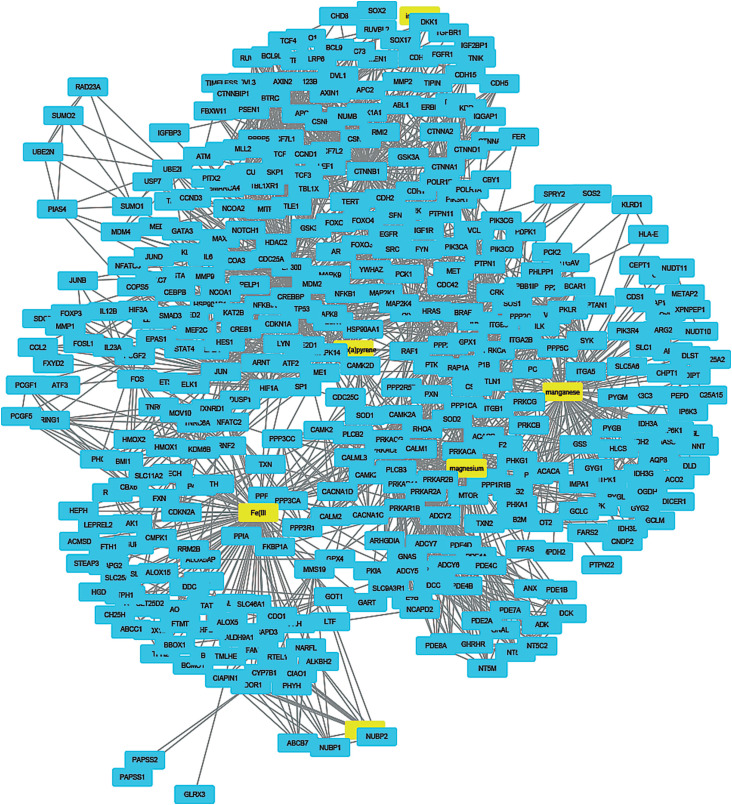
Cluster analysis of the major CPI-PPI network showing the association of cluster 14 with MIOs, IEs, and PAHs (yellow). It is composed of 432 nodes and 2,520 edges, with Ci = 1,164. The associated constituents are S, Mn, Mg, Fe, Fe_2_O_3_ and benzo[a]pyrene.

**Figure 6:**
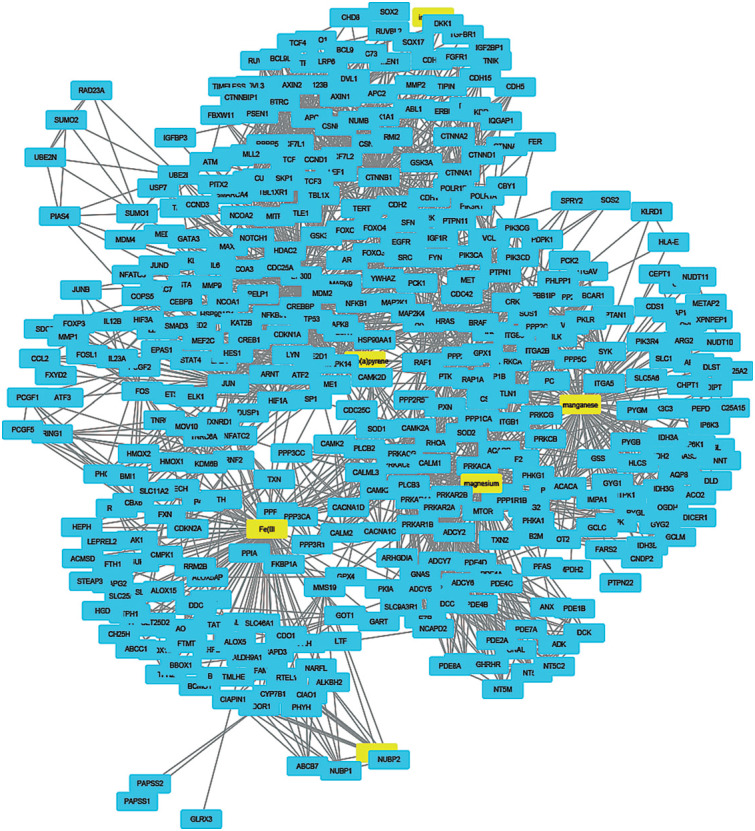
Cluster analysis of the major CPI-PPI network showing the association of cluster 9 with IEs and PAHs (yellow). It is composed of 249 nodes and 2,180 edges, with Ci = 17,44. The associated compounds are S, Cr, Ti, and benzo[a]pyrene.

**Figure 7:**
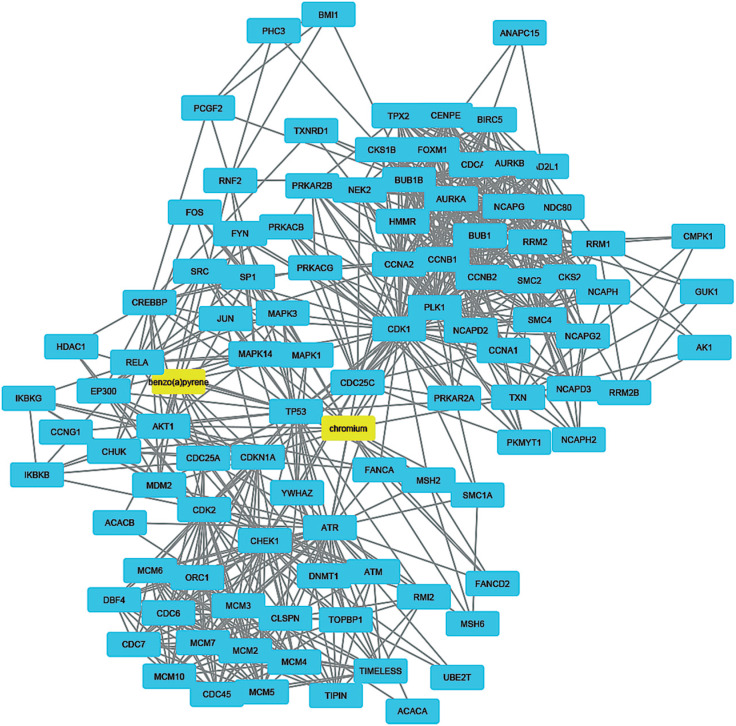
Cluster analysis of the major CPI-PPI network showing the association of cluster 12 with IEs and PAHs (yellow). It is composed of 102 nodes and 741 edges, with Ci = 14,388. The associated compounds are Cr and benzo[a]pyrene.

**Figure 8:**
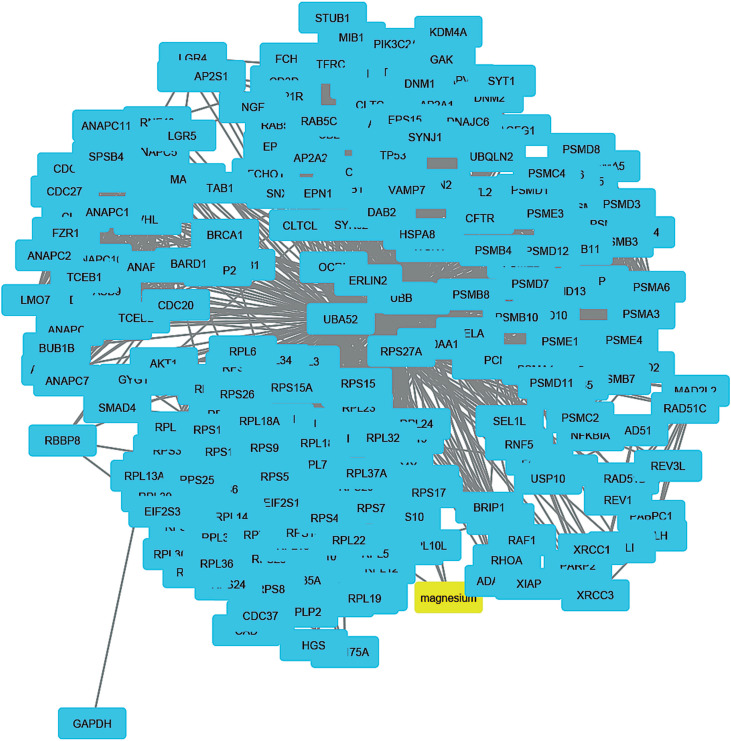
Cluster analysis of the major CPI-PPI network showing association of cluster 2 with IEs (yellow). It is composed of 250 nodes and 5,976 edges, with Ci = 47,618, associated with Mg.

**Figure 9:**
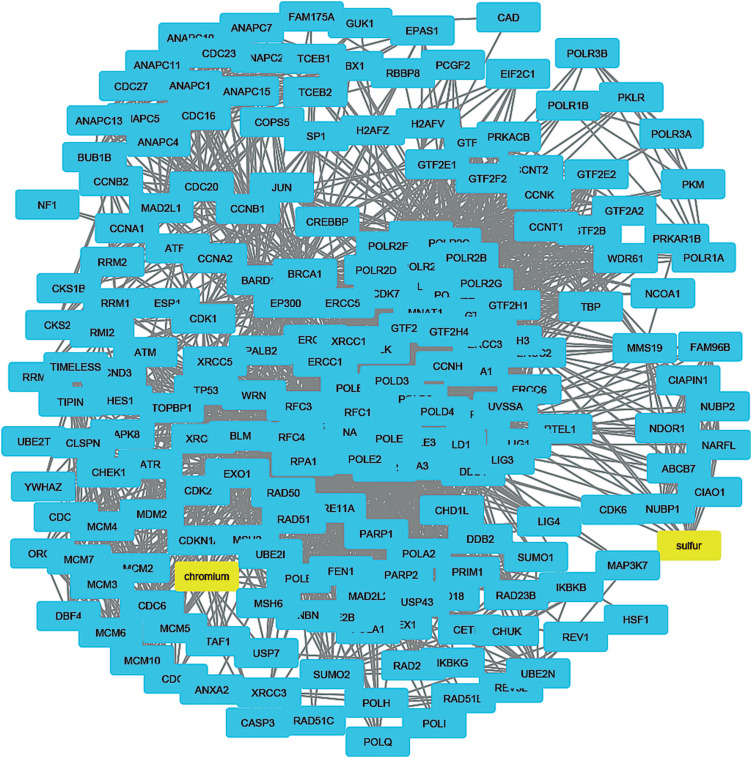
Cluster analysis of the major CPI-PPI network showing association of cluster 4 with IEs (yellow). It is composed of 208 nodes and 3,134 edges, with Ci = 2,999, associated with Cr and S.

To understand how coal chemical constituents interact with cell cycle processes, we identified the modules in the main CPI-PPI network using the MCODE program. From these analyses, we obtained eight significant modules related to cell cycle processes (Figures 2 - 9). Clusters 6 (Figure 2), 11(Figure 3), 13 (Figure 4), and 14 (Figure 5) are associated with MIOs, IEs and PAHs; clusters 9 (Figure 6) and 12 (Figure 7) appear to be associated with IEs and PAHs; finally clusters 2 (Figure 8) and 4 (Figure 9) are associated with IEs only. The analysis revealed 15 common proteins associated with different cell cycle processes.

**Figure 10:**
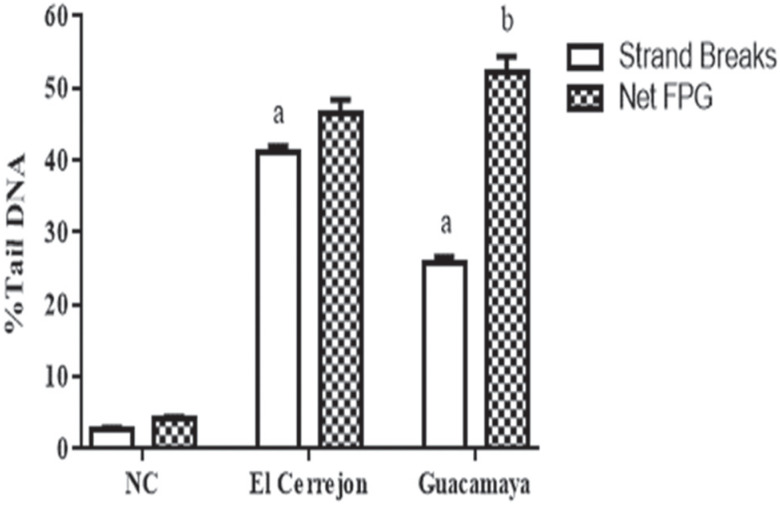
Percent tail DNA in the alkaline comet assay (strand breaks in white) and oxidized purines (grid) in a modified comet assay with FPG in V79 cells under 24 h exposure with El Cerrejon and Gucamaya coal. a) Statistically significant differences in relation negative control (NC) without enzyme P <0.05. b) Statistically significant differences in relation to the same sample group with an enzyme. The results are shown as the mean ± SEM.

The DNA damage induced by El Cerrejón and Guacamaya coal was determined by the modified alkaline high-throughput version of the comet assay and evaluated by the % tail DNA. The results of the comet assay showed statistically significant differences in relation to the negative control (NC) without enzyme (P <0.05) and the % DNA tail increase. Additionally, the results of the modified comet assay showed a statistically significant difference when compared with the same sample group (P < 0.05) (Figure 10).

## Discussion

Ubiquitin (UBC) and two ubiquitin-coding genes (UBA52 and RPS27A) demonstrated the highest node degree and betweenness values, thus representing highly central proteins inside the network (Feltes *et al.*, 2013). UBC is a small 76-amino acid protein that is involved in several different pathways within the cell, including the clearing of damaged/misfolded proteins during proteotoxic stress (Bianchi *et al.*, 2015). UBC genes are upregulated in response oxidative stress (Lee and Ryu, 2017), thereby increasing cellular UBC above threshold levels and conferring resistance to oxidative damage.

### Systemic effects of MIOs, IEs and PAHs in the cell cycle and DNA damage

Tables 2–4 show the results of the GO analysis for each cluster and the cell cycle process categories. The main biological processes linked to clusters 6, 11, 13 and 14 included the following: (i) cell cycle process, (ii) mitotic cell cycle, (iii) cell cycle, (iv) cell cycle checkpoint, (v) regulation of cell cycle and (vi) cell cycle arrest (Table 2). Interestingly, DNA repair bioprocesses were found in this module only in co-occurrence with MIOs, IEs and PAHs. The particular combination of these compounds is associated with increased DNA damage in cell systems *in vitro* (Leon-Mejia *et al.*, 2016) and human populations in coal mining environments (Leon-Mejia *et al.*, 2011). The primary mechanism proposed for these effects involves oxidative damage through the production of ROS (Valko *et al.*, 2006). In this regard, within the same module, proteins regulated by oxidative stress inside the cell were identified as bottlenecks (AKT, APP, JUN and CREBBP). While AKT has been reported to be regulated by oxidative stress for cell survival (Wang *et al.*, 2000), several studies have indicated that oxidative stress participates in events that enhance amyloidogenic APP processing in neurons (Lin and Beal, 2006; Mouton-Liger *et al.*, 2012) and in events that affect cerebrovascular endothelial APP processing (Muche *et al.*, 2017). ROS-facilitated protein phosphorylation can also lead to kinase-mediated activation of transcription factors, such as the JUN group (Nathan and Cunningham-Bussel, 2013), affecting cell cycle progression by their ability to regulate the expression and function of cell cycle regulators such as cyclins (Schreiber *et al.*, 1999; Chiba *et al.*, 2017), and apoptosis (Meixner *et al.*, 2010). Together with JUN, CREBBP is also involved in cell division and cell proliferation, and it is upregulated by the oxidative stress response in retinoblastoma cells (Meixner *et al.*, 2010).

**Table 2:** Major cell cycle bioprocesses in clusters 6, 11, 13 and 14 associated with MIOs, IEs and PAHs.

GO ID	p-value	corr p-value	k*	n#	Description	Genes in test set
22402	1,64E-22	9,74E-21	77	582	cell cycle process	APP|CDKN1A|CETN2|CLTC|UBE2D1|PSMD8|PSMD9|PPP3CA|PSMD7|PSMD4|PSMD2|PSMD3|PSMD1|AKT1|IL12B|NBN|POLE|APC2|H2AFX|CDC25A|DNM2|PSMA5|PSMA6|DNAJC2|PSMA3|ADAM17|PSMA4|PSMA1|PSME3|PSME1|PSME2|TP53|PSMD10|PSMD12|PSMD11|RGS14|PSMD13|CUL2|THBS1|EGFR|PSMB10|PSMB6|PSMB7|PSMB4|C6|PSMB5|PSMB2|PSMB3|POLD1|PSMB1|CLTCL1|APBB1|UBE2I|TGFB1|SMAD3|VDR|RPA1|MRE11A|CDC6|HSPA2|PSMB8|MAPK12|PSMB9|PPP5C|RAD50|PSMC6|PSMC3|APC|IL8|PSMC4|PSMC1|PSMC2|CDK2|MDM2|CTNNB1|CALR|SUGT1
278	9,54E-21	5,08E-19	60	380	mitotic cell cycle	APP|CDKN1A|CETN2|CLTC|UBE2D1|PSMD8|PSMD9|PPP3CA|PSMD7|PSMD4|PSMD2|PSMD3|PSMD1|AKT1|POLE|APC2|CDC25A|DNM2|PSMA5|PSMA6|DNAJC2|PSMA3|ADAM17|PSMA4|PSMA1|PSME3|PSME1|PSME2|PSMD10|PSMD12|PSMD11|RGS14|PSMD13|CUL2|EGFR|PSMB10|PSMB6|PSMB7|PSMB4|C6|PSMB5|PSMB2|PSMB3|POLD1|PSMB1|CLTCL1|UBE2I|CDC6|PSMB8|PSMB9|PPP5C|PSMC6|PSMC3|APC|PSMC4|PSMC1|PSMC2|CDK2|MDM2|SUGT1
7049	3,12E-18	1,43E-16	84	794	cell cycle	APP|CDKN1A|STEAP3|CCNH|CETN2|CLTC|UBE2D1|PSMD8|PSMD9|PPP3CA|PSMD7|PSMD4|PSMD2|PSMD3|PSMD1|AKT1|IL12B|EP300|NBN|POLE|APC2|ANXA1|H2AFX|CDC25A|DNM2|PSMA5|GAK|PSMA6|DNAJC2|PSMA3|ADAM17|PSMA4|PSMA1|PSME3|PSME1|PSME2|TP53|PSMD10|PSMD12|PSMD11|RGS14|PSMD13|CUL2|THBS1|EGFR|PSMB10|PSMB6|PSMB7|PSMB4|C6|PSMB5|PSMB2|PSMB3|POLD1|PSMB1|CLTCL1|APBB1|UBE2I|TGFB1|SMAD3|VDR|RPA1|MRE11A|CDC6|HSPA2|PSMB8|MAPK12|PSMB9|CYLD|PPP5C|RAD50|PSMC6|PSMC3|APC|IL8|PSMC4|PSMC1|PSMC2|CDK2|MDM2|CTNNB1|REN|CALR|SUGT1
22402	4,62E-06	1,46E-04	22	582	cell cycle process	CDKN1A|NPM1|UBE2I|CDKN2A|CETN2|PLK1|H2AFX|NCAPG|SMC1A|MLH1|CENPA|NDC80|SMC2|MSH6|PPP2CA|POLA1|PPP5C|MSH2|FANCD2|CDK1|NBN|TP53
75	5,61E-06	1,73E-04	11	107	cell cycle checkpoint	CDKN1A|MSH2|CDKN2A|PLK1|H2AFX|CDK1|NBN|PPP2R5C|SMC1A|TP53|ATR
6281	7,00E-12	4,71E-10	22	298	DNA repair	POLQ|FEN1|PARP1|PRKDC|PARP2|H2AFX|RAD23A|SMC1A|MLH1|RAD23B|DDB2|MSH6|POLB|POLA1|MSH2|SUMO1|FANCD2|APEX1|UBE2N|NBN|TP53|ATR
51726	1,37E-05	1,78E-04	19	446	regulation of cell cycle	MAP2K1|JUN|CREBBP|CDKN1A|HDAC2|NGF|TNF|CYLD|KAT2B|IL1A|ADAM17|IFNG|CDC37|CASP3|IL1B|IL12B|AKT1|HRAS|MAP2K6
51726	5,25E-11	1,98E-09	48	447	regulation of cell cycle	CDS1|CDKN1A|HDAC2|TRRAP|HDAC1|CITED2|CUL1|ILK|FOXO4|ETS1|EGFR|SOX2|CCND3|CCND1|CDH1|AKT1|IL12B|SFN|PRKACA|BTRC|JUNB|HRAS|MEN1|APC2|TCF7L2|JUN|CREBBP|MAP2K1|TIPIN|SMAD3|CDKN2A|GSS|INSR|PTPN11|CDC25C|CDC25A|SMARCA4|FOSL1|KAT2B|COPS5|APC|PKIA|MDM2|TIMELESS|ATM|TCF4|TCF3|TP53
22402	9,37E-06	1,83E-04	46	583	cell cycle process	CAMK2B|CDKN1A|NCAPG2|CUL1|UBE2D1|ILK|FOXO4|EGFR|SOX2|PPP2CA|PPP3CA|CCND1|CDH1|RUVBL1|ABL1|AKT1|IL12B|BTRC|HRAS|MEN1|SKP1|APC2|TCF7L2|MAP2K1|TIPIN|UBE2I|SMAD3|CSNK1A1|CDKN2A|GSS|CDC25C|CDC25A|KAT2B|PPP5C|APC|MDM2|TIMELESS|CTNNB1|NCAPD2|MDM4|ATM|TCF4|NCAPD3|TCF3|TP53|TAF1
7049	2,41E-05	4,31E-04	55	795	cell cycle	CDKN1A|STEAP3|NCAPG2|UBE2D1|ILK|CDC73|SOX2|PPP3CA|CCND1|CDH1|RUVBL1|AKT1|IL12B|EP300|BTRC|HRAS|MEN1|SKP1|APC2|MAP2K1|TIPIN|ANXA1|DUSP1|FBXW11|CDC25C|CDC25A|KAT2B|TIMELESS|TP53|CDS1|CAMK2B|CUL1|FOXO4|EGFR|RNF2|PPP2CA|ABL1|TCF7L2|UBE2I|SMAD3|CSNK1A1|CDKN2A|GSS|PPP1CA|PPP5C|APC|MDM2|CTNNB1|NCAPD2|MDM4|ATM|TCF4|NCAPD3|TCF3|TAF1
7050	2,63E-05	4,64E-04	18	109	cell cycle arrest	TCF7L2|CDKN1A|MAP2K1|SMAD3|CDKN2A|GSS|CUL1|ILK|FOXO4|SOX2|KAT2B|APC|IL12B|ATM|TCF4|TP53|HRAS|MEN1

# total number of nodes in the gene ontology (GO) annotation;

* number of nodes related to a given GO in the network.

**Table 3:** Major cell cycle bioprocesses in clusters 9 and 12 associated with IEs and PAHs.

GO ID	p-value	corr p-	k*	n#	Description	Genes in test set
22402	2,71E-34	3,52E-31	67	582	cell cycle process	UBE2D1|BUB1B|CDC20|PPP3CA|CDC23|EXO1|CHEK1|CDC27|IL12B|AKT1|NEK2|NBN|HRAS|TIPIN|ANAPC7|H2AFX|CDC25C|RAD51B|MSH6|CCNA2|CCNA1|RAD51C|MSH2|IFNG|CKS2|TIMELESS|ANAPC4|BIRC5|ANAPC5|TP53|ANAPC1|ANAPC2|ANAPC13|BLM|CUL5|CUL2|NCAPG|CDCA8|PKMYT1|CENPA|THBS1|ANAPC10|EGFR|AURKB|ANAPC11|AURKA|CCNB2|CCNB1|FZR1|BUB1|BARD1|UBE2I|TGFB1|PLK1|MRE11A|CDC6|MLH1|NDC80|TPX2|CENPE|RAD50|RAD51|CDC16|CDK2|CDK1|ATM|MAD2L1
7049	3,93E-33	3,40E-30	75	794	cell cycle	UBE2D1|BUB1B|FOXM1|CKS1B|CDC20|PPP3CA|CDC23|EXO1|CHEK1|CDC27|IL12B|AKT1|EP300|NEK2|NBN|HRAS|TIPIN|ANAPC7|H2AFX|CDC25C|RAD51B|MSH6|CCNA2|CCNA1|RAD51C|MSH2|IFNG|CKS2|TIMELESS|ANAPC4|BIRC5|ANAPC5|TP53|ANAPC1|ANAPC2|ANAPC13|BLM|CUL5|CUL2|NCAPG|CDCA8|PKMYT1|CENPA|THBS1|ANAPC10|EGFR|AURKB|ANAPC11|AURKA|CCNB2|CCNB1|FZR1|CDC45|MAPK1|CLSPN|BUB1|MAPK3|BARD1|UBE2I|TGFB1|PLK1|MRE11A|CDC6|MLH1|NDC80|TPX2|CENPE|RAD50|RAD51|CDC16|CDK2|CDK1|ATM|ATR|MAD2L1
22403	2,04E-31	1,06E-28	57	435	cell cycle phase	BUB1B|CDC20|PPP3CA|CDC23|EXO1|CHEK1|CDC27|AKT1|NEK2|NBN|TIPIN|ANAPC7|H2AFX|CDC25C|RAD51B|MSH6|CCNA2|CCNA1|RAD51C|CKS2|TIMELESS|ANAPC4|BIRC5|ANAPC5|ANAPC1|ANAPC2|ANAPC13|BLM|CUL5|CUL2|NCAPG|CDCA8|PKMYT1|ANAPC10|EGFR|AURKB|ANAPC11|AURKA|CCNB2|CCNB1|FZR1|BUB1|UBE2I|PLK1|MRE11A|CDC6|MLH1|NDC80|TPX2|CENPE|RAD50|RAD51|CDC16|CDK2|CDK1|ATM|MAD2L1
51726	1,03E-27	3,84E-25	54	446	regulation of cell cycle	BUB1B|FOXM1|CKS1B|CDC23|CHEK1|IL12B|AKT1|NEK2|NBN|PRKACA|HRAS|TIPIN|H2AFX|CDC25C|CCNA2|MSH2|IFNG|CDC37|CKS2|TIMELESS|BIRC5|TP53|ANAPC2|BLM|CUL5|CUL2|PKMYT1|THBS1|ANAPC10|EGFR|BRIP1|CCNB1|FZR1|CDC45|RBBP8|CLSPN|BUB1|BARD1|JUN|CREBBP|TGFB1|PLK1|MRE11A|CDC6|TPX2|CENPE|COPS5|CDC16|FAM175A|CDK2|CDK1|ATM|ATR|MAD2L1
278	6,00E-23	8,21E-21	46	380	mitotic cell cycle	ANAPC13|BLM|CUL5|CUL2|NCAPG|CDCA8|UBE2D1|BUB1B|PKMYT1|CENPA|ANAPC10|EGFR|AURKB|ANAPC11|AURKA|CDC20|PPP3CA|CCNB2|CCNB1|FZR1|CDC23|CDC27|AKT1|NEK2|BUB1|TIPIN|UBE2I|ANAPC7|PLK1|CDC6|CDC25C|NDC80|CCNA2|TPX2|CENPE|CCNA1|CDC16|CDK2|TIMELESS|CDK1|ANAPC4|BIRC5|ANAPC5|ANAPC1|MAD2L1|ANAPC2
87	1,10E-22	1,36E-20	38	239	M phase of mitotic cell cycle	ANAPC13|NCAPG|CDCA8|BUB1B|PKMYT1|ANAPC10|AURKB|ANAPC11|AURKA|CDC20|CCNB2|CCNB1|FZR1|CDC23|CDC27|NEK2|BUB1|TIPIN|UBE2I|ANAPC7|PLK1|CDC6|CDC25C|NDC80|CCNA2|TPX2|CENPE|CCNA1|CDC16|CDK2|TIMELESS|CDK1|ANAPC4|BIRC5|ANAPC5|ANAPC1|MAD2L1|ANAPC2
75	8,32E-21	6,55E-19	27	107	cell cycle checkpoint	BLM|BUB1B|BRIP1|CCNB1|FZR1|CDC45|CHEK1|RBBP8|NBN|CLSPN|HRAS|BUB1|TIPIN|TGFB1|PLK1|H2AFX|CDC6|CCNA2|CENPE|MSH2|FAM175A|CDK1|BIRC5|ATM|TP53|ATR|MAD2L1
7346	1,07E-11	3,66E-10	24	174	regulation of mitotic cell cycle	TGFB1|PLK1|BUB1B|CDC6|CDC25C|PKMYT1|ANAPC10|EGFR|CCNA2|TPX2|CENPE|CCNB1|CDC23|CDC16|CDK2|CDK1|BIRC5|ATM|NEK2|NBN|TP53|HRAS|BUB1|MAD2L1
10564	1,19E-08	2,53E-07	19	138	regulation of cell cycle process	TIPIN|CREBBP|TGFB1|MRE11A|CDC25C|PKMYT1|FOXM1|ANAPC10|TPX2|CENPE|CCNB1|FZR1|CDC23|CDC16|TIMELESS|BIRC5|ATM|NEK2|BUB1
7093	3,57E-07	5,87E-06	12	52	mitotic cell cycle checkpoint	CCNA2|CENPE|CCNB1|TGFB1|CDK1|BUB1B|ATM|NBN|TP53|HRAS|BUB1|MAD2L1
51327	1,15E-06	1,75E-05	15	102	M phase of meiotic cell cycle	H2AFX|MRE11A|MLH1|RAD51B|MSH6|CCNA1|RAD50|RAD51C|RAD51|EXO1|CHEK1|CKS2|ATM|NEK2|NBN
51329	1,15E-06	1,75E-05	15	102	interphase of mitotic cell cycle	BLM|CUL5|CUL2|CDC6|CDC25C|ANAPC10|EGFR|PPP3CA|CCNB1|CDC23|CDK2|AKT1|ANAPC4|BIRC5|ANAPC5
51321	1,33E-06	1,99E-05	15	103	meiotic cell cycle	H2AFX|MRE11A|MLH1|RAD51B|MSH6|CCNA1|RAD50|RAD51C|RAD51|EXO1|CHEK1|CKS2|ATM|NEK2|NBN
7049	4,53E-45	6,63E-42	59	794	cell cycle	CDKN1A|MCM7|NCAPG2|BUB1B|FOXM1|SMC4|SMC2|CKS1B|CHEK1|EP300|AKT1|NEK2|TIPIN|CDC25C|SMC1A|CDC25A|MSH6|CCNA2|CCNA1|DBF4|MSH2|FANCD2|TIMELESS|MCM3|CKS2|BIRC5|MCM6|TP53|MCM2|NCAPG|CDCA8|PKMYT1|NCAPH|RNF2|AURKB|AURKA|CCNB2|CCNB1|CDC45|MAPK1|CLSPN|BUB1|MAPK3|PLK1|FANCA|CDC7|CDC6|NDC80|TPX2|CENPE|CDK2|CCNG1|MDM2|CDK1|ATM|NCAPD2|NCAPD3|ATR|MAD2L1
22403	7,21E-37	5,27E-34	44	435	cell cycle phase	CDKN1A|NCAPG2|BUB1B|NCAPG|CDCA8|PKMYT1|SMC4|NCAPH|AURKB|SMC2|AURKA|CCNB2|CCNB1|CHEK1|AKT1|NEK2|BUB1|TIPIN|PLK1|FANCA|CDC7|CDC6|CDC25C|SMC1A|CDC25A|NDC80|MSH6|CCNA2|TPX2|CCNA1|CENPE|DBF4|FANCD2|CDK2|CCNG1|TIMELESS|MDM2|CDK1|CKS2|BIRC5|ATM|NCAPD2|NCAPD3|MAD2L1
22402	5,73E-34	2,79E-31	46	582	cell cycle process	CDKN1A|NCAPG2|BUB1B|NCAPG|CDCA8|PKMYT1|SMC4|NCAPH|AURKB|SMC2|AURKA|CCNB2|CCNB1|CHEK1|AKT1|NEK2|BUB1|TIPIN|PLK1|FANCA|CDC7|CDC6|CDC25C|SMC1A|CDC25A|NDC80|MSH6|CCNA2|TPX2|CCNA1|CENPE|DBF4|MSH2|FANCD2|CDK2|CCNG1|TIMELESS|MDM2|CDK1|CKS2|BIRC5|ATM|NCAPD2|NCAPD3|TP53|MAD2L1
278	4,13E-32	1,21E-29	39	380	mitotic cell cycle	CDKN1A|NCAPG2|BUB1B|NCAPG|CDCA8|PKMYT1|SMC4|NCAPH|RNF2|AURKB|SMC2|AURKA|CCNB2|CCNB1|AKT1|NEK2|BUB1|TIPIN|PLK1|CDC7|CDC6|CDC25C|SMC1A|CDC25A|NDC80|CCNA2|TPX2|CCNA1|CENPE|DBF4|CDK2|CCNG1|TIMELESS|MDM2|CDK1|BIRC5|NCAPD2|NCAPD3|MAD2L1
87	1,23E-30	3,01E-28	33	239	M phase of mitotic cell cycle	NCAPG2|BUB1B|NCAPG|CDCA8|PKMYT1|SMC4|NCAPH|AURKB|SMC2|AURKA|CCNB2|CCNB1|NEK2|BUB1|TIPIN|PLK1|CDC6|CDC25C|SMC1A|CDC25A|NDC80|CCNA2|TPX2|CCNA1|CENPE|CDK2|CCNG1|TIMELESS|CDK1|BIRC5|NCAPD2|NCAPD3|MAD2L1
51726	1,02E-26	1,36E-24	37	446	regulation of cell cycle	CDKN1A|HDAC1|BUB1B|PKMYT1|FOXM1|CKS1B|CCNB1|CDC45|CHEK1|AKT1|NEK2|CLSPN|BUB1|TIPIN|JUN|CREBBP|PLK1|CDC7|CDC6|CDC25C|SMC1A|CDC25A|CCNA2|TPX2|CENPE|MSH2|CDK2|CCNG1|TIMELESS|MDM2|CDK1|CKS2|BIRC5|ATM|TP53|ATR|MAD2L1
75	2,29E-21	2,57E-19	21	107	cell cycle checkpoint	TIPIN|CDKN1A|PLK1|BUB1B|CDC6|SMC1A|CCNA2|CENPE|CCNB1|CDC45|MSH2|CHEK1|CCNG1|CDK1|BIRC5|ATM|CLSPN|TP53|BUB1|ATR|MAD2L1
7346	2,52E-15	2,30E-13	20	174	regulation of mitotic cell cycle	CDKN1A|PLK1|BUB1B|CDC6|CDC25C|PKMYT1|CCNA2|TPX2|CENPE|CCNB1|CDK2|CCNG1|MDM2|CDK1|BIRC5|NEK2|ATM|TP53|BUB1|MAD2L1
10564	4,70E-13	3,44E-11	17	138	regulation of cell cycle process	TIPIN|CREBBP|CDKN1A|CDC7|CDC25C|PKMYT1|FOXM1|SMC1A|TPX2|CENPE|CCNB1|TIMELESS|MDM2|BIRC5|NEK2|ATM|BUB1
7093	4,22E-10	1,81E-08	11	52	mitotic cell cycle checkpoint	CCNA2|CENPE|CCNB1|CDKN1A|CCNG1|CDK1|BUB1B|ATM|TP53|BUB1|MAD2L1
51329	9,39E-07	2,41E-05	11	102	interphase of mitotic cell cycle	CCNB1|CDKN1A|DBF4|CDK2|MDM2|AKT1|BIRC5|CDC7|CDC6|CDC25C|CDC25A

# total number of nodes in the gene ontology (GO) annotation;

* number of nodes related to a given GO in the network.

**Table 4:** Major cell cycle bioprocesses in clusters 2 and 4 associated with IEs.

GO ID	p-value	corr p-	k*	n#	Description	Genes in test set
278	6,09E-38	2,63E-36	60	380	mitotic cell cycle	CLTC|BUB1B|PSMD8|CDC20|PSMD9|PSMD7|CDC23|PSMD2|PSMD3|CDC27|PSMD1|AKT1|ANAPC7|RPS6|DNM2|PSMA5|PSMA6|PSMA3|ADAM17|PSMA4|PSMA1|PSME3|PSME1|RPL24|PSME2|ANAPC4|ANAPC5|ANAPC1|ANAPC2|PSMD10|ANAPC13|PSMD12|PSMD11|CUL5|PSMD13|CUL2|ANAPC10|PSMB10|ANAPC11|PSMB6|PSMB7|PSMB4|CCNB1|FZR1|PSMB5|PSMB2|PSMB3|PSMB1|CLTCL1|PSMB8|PSMB9|MAD2L2|PSMC6|PSMC3|PSMC4|PSMC1|CDC16|PSMC2|MDM2|MAD2L1
22402	4,93E-32	1,84E-30	65	582	cell cycle process	CLTC|BUB1B|PSMD8|CDC20|PSMD9|PSMD7|CDC23|PSMD2|PSMD3|CDC27|PSMD1|AKT1|ANAPC7|RPS6|DNM2|RAD51B|PSMA5|PSMA6|PSMA3|ADAM17|RAD51C|PSMA4|PSMA1|PSME3|PSME1|RPL24|PSME2|ANAPC4|ANAPC5|TP53|ANAPC1|ANAPC2|PSMD10|ANAPC13|PSMD12|PSMD11|CUL5|PSMD13|CUL2|ANAPC10|PSMB10|ANAPC11|PSMB6|PSMB7|PSMB4|CCNB1|FZR1|PSMB5|PSMB2|PSMB3|PSMB1|CLTCL1|BARD1|PSMB8|PSMB9|MAD2L2|PSMC6|RAD51|PSMC3|PSMC4|PSMC1|CDC16|PSMC2|MDM2|MAD2L1
7049	1,12E-25	3,45E-24	67	794	cell cycle	CLTC|BUB1B|BRCA1|PSMD8|CDC20|PSMD9|PSMD7|CDC23|PSMD2|PSMD3|CDC27|PSMD1|AKT1|ANAPC7|RPS6|DNM2|RAD51B|PSMA5|GAK|PSMA6|PSMA3|ADAM17|RAD51C|PSMA4|PSMA1|PSME3|PSME1|RPL24|PSME2|ANAPC4|ANAPC5|TP53|ANAPC1|ANAPC2|PSMD10|ANAPC13|PSMD12|PSMD11|CUL5|PSMD13|CUL2|ANAPC10|PSMB10|ANAPC11|PSMB6|PSMB7|PSMB4|CCNB1|FZR1|PSMB5|PSMB2|PSMB3|PSMB1|CLTCL1|BARD1|PSMB8|PSMB9|MAD2L2|PSMC6|RAD51|PSMC3|PSMC4|PSMC1|CDC16|PSMC2|MDM2|MAD2L1
22403	3,84E-07	8,30E-06	30	435	cell cycle phase	ANAPC13|CUL5|CLTC|CUL2|BUB1B|ANAPC10|ANAPC11|CDC20|CCNB1|FZR1|CDC23|CDC27|CLTCL1|AKT1|ANAPC7|RPS6|DNM2|RAD51B|MAD2L2|ADAM17|RAD51C|RAD51|CDC16|MDM2|RPL24|ANAPC4|ANAPC5|ANAPC1|MAD2L1|ANAPC2
87	5,19E-06	1,03E-04	21	239	M phase of mitotic cell cycle	ANAPC13|ANAPC7|CLTC|RPS6|BUB1B|ANAPC10|ANAPC11|CDC20|MAD2L2|CCNB1|FZR1|CDC23|CDC27|CDC16|CLTCL1|RPL24|ANAPC4|ANAPC5|ANAPC1|MAD2L1|ANAPC2
7049	5,73E-35	4,37E-33	71	794	cell cycle	CCNK|CDKN1A|CCNT2|CCNT1|MCM7|CCNH|CETN2|BUB1B|BRCA1|CKS1B|CDC20|CDC23|EXO1|CHEK1|CDC27|EP300|NBN|POLK|POLE|TIPIN|LIG1|ANAPC7|LIG4|LIG3|RAD51B|MSH6|CCNA2|CCNA1|RAD51C|DBF4|MSH2|CKS2|TIMELESS|MCM3|ANAPC4|ANAPC5|MCM6|TP53|ANAPC1|ANAPC2|MCM2|ANAPC13|BLM|ANAPC10|ANAPC11|CCNB2|CCNB1|CDC45|POLD1|CLSPN|BARD1|UBE2I|UBE2B|RPA1|MRE11A|CDC7|CDC6|MAD2L2|POLA1|RAD50|CDK6|RAD51|CDC16|CDK2|MDM2|CDK1|ATM|MNAT1|ATR|MAD2L1|TAF1
22403	2,64E-31	1,79E-29	53	435	cell cycle phase	CCNK|CDKN1A|CETN2|BUB1B|CDC20|CDC23|EXO1|CHEK1|CDC27|NBN|POLK|POLE|TIPIN|ANAPC7|LIG3|RAD51B|MSH6|CCNA2|CCNA1|RAD51C|DBF4|CKS2|TIMELESS|ANAPC4|ANAPC5|ANAPC1|ANAPC2|ANAPC13|BLM|ANAPC10|ANAPC11|CCNB2|CCNB1|POLD1|UBE2I|UBE2B|RPA1|MRE11A|CDC7|CDC6|MAD2L2|POLA1|RAD50|CDK6|RAD51|CDC16|CDK2|MDM2|CDK1|ATM|MNAT1|MAD2L1|TAF1
22402	7,25E-28	4,15E-26	56	582	cell cycle process	CCNK|CDKN1A|CETN2|BUB1B|CDC20|CDC23|EXO1|CHEK1|CDC27|NBN|POLK|POLE|TIPIN|ANAPC7|LIG3|RAD51B|MSH6|CCNA2|CCNA1|RAD51C|DBF4|MSH2|CKS2|TIMELESS|ANAPC4|ANAPC5|TP53|ANAPC1|ANAPC2|ANAPC13|BLM|ANAPC10|ANAPC11|CCNB2|CCNB1|POLD1|BARD1|UBE2I|UBE2B|RPA1|MRE11A|CDC7|CDC6|MAD2L2|POLA1|RAD50|CDK6|RAD51|CDC16|CDK2|MDM2|CDK1|ATM|MNAT1|MAD2L1|TAF1
51726	1,75E-27	9,46E-26	50	446	regulation of cell cycle	CCNK|CDKN1A|CCNT2|CCNT1|BUB1B|BRCA1|CKS1B|CCND3|CDC23|CASP3|CHEK1|NBN|TIPIN|DDB1|CCNA2|MSH2|CKS2|TIMELESS|TP53|ANAPC2|BLM|ANAPC10|BRIP1|CCNB1|CDC45|RBBP8|CLSPN|BARD1|JUN|CREBBP|UBE2B|MRE11A|CDC7|GTF2H1|CDC6|MAD2L2|CDK7|COPS5|CDK6|ERCC3|CDC16|FAM175A|CDK2|ERCC2|MDM2|CDK1|ATM|MNAT1|ATR|MAD2L1
75	5,24E-20	2,13E-18	25	107	cell cycle checkpoint	CDKN1A|BLM|BUB1B|BRCA1|BRIP1|CCNB1|CDC45|CHEK1|RBBP8|NBN|CLSPN|TIPIN|CDC6|DDB1|MAD2L2|CCNA2|MSH2|ERCC3|FAM175A|ERCC2|CDK1|ATM|TP53|ATR|MAD2L1
278	4,71E-19	1,88E-17	39	380	mitotic cell cycle	ANAPC13|CCNK|CDKN1A|BLM|CETN2|BUB1B|ANAPC10|ANAPC11|CDC20|CCNB2|CCNB1|CDC23|POLD1|CDC27|POLK|POLE|TIPIN|UBE2I|ANAPC7|CDC7|CDC6|MAD2L2|CCNA2|POLA1|CCNA1|CDK6|DBF4|CDC16|CDK2|TIMELESS|MDM2|CDK1|ANAPC4|ANAPC5|MNAT1|ANAPC1|MAD2L1|ANAPC2|TAF1
87	3,12E-14	1,00E-12	28	239	M phase of mitotic cell cycle	ANAPC13|CCNK|CETN2|BUB1B|ANAPC10|ANAPC11|CDC20|CCNB2|CCNB1|CDC23|CDC27|POLK|TIPIN|UBE2I|ANAPC7|CDC6|MAD2L2|CCNA2|CCNA1|CDC16|CDK2|TIMELESS|CDK1|ANAPC4|ANAPC5|ANAPC1|MAD2L1|ANAPC2
51329	2,90E-11	6,99E-10	18	102	interphase of mitotic cell cycle	CDKN1A|BLM|CDC7|CDC6|ANAPC10|POLA1|CCNB1|CDC23|CDK6|DBF4|POLD1|CDK2|MDM2|ANAPC4|ANAPC5|MNAT1|POLE|TAF1
51327	1,02E-07	1,44E-06	15	102	M phase of meiotic cell cycle	UBE2B|RPA1|MRE11A|LIG3|RAD51B|MSH6|CCNA1|RAD50|RAD51C|RAD51|EXO1|CHEK1|CKS2|ATM|NBN
51321	1,18E-07	1,65E-06	15	103	meiotic cell cycle	UBE2B|RPA1|MRE11A|LIG3|RAD51B|MSH6|CCNA1|RAD50|RAD51C|RAD51|EXO1|CHEK1|CKS2|ATM|NBN
10564	8,19E-06	9,75E-05	15	138	regulation of cell cycle process	TIPIN|CREBBP|CDKN1A|UBE2B|MRE11A|CDC7|BRCA1|ANAPC10|MAD2L2|CCNB1|CDC23|CDC16|TIMELESS|MDM2|ATM
7093	2,08E-05	2,38E-04	10	52	mitotic cell cycle checkpoint	MAD2L2|CCNA2|CDKN1A|CCNB1|CDK1|BUB1B|ATM|NBN|TP53|MAD2L1
7346	2,63E-05	2,95E-04	16	174	regulation of mitotic cell cycle	CDKN1A|BUB1B|CDC6|ANAPC10|MAD2L2|CCNA2|CCNB1|CDC23|CDC16|CDK2|MDM2|CDK1|ATM|NBN|TP53|MAD2L1
86	9,00E-05	9,82E-04	7	21	G2/M transition of mitotic cell cycle	CDKN1A|CCNB1|CDK2|ANAPC4|ANAPC5|ANAPC10|TAF1

# total number of nodes in the gene ontology (GO) annotation;

* number of nodes related to a given GO in the netwo

Oxidative stress can generate alterations in the progression of the cell cycle (blockage and/or delay), as well as structural dysfunction in several proteins. DNA-integrity checkpoints G1/S and G2/M, and M/A transitions determine cell cycle delays (Rieder, 2011) depending on the cyclin-dependent kinase (Cdk)/cyclin system, such as Cdk1/cyclin B1, which drives the progression from G2 to the mitotic phase (Pearce and Humphrey, 2001). The protein kinases ataxia-telangiectasia mutated (ATM) and ATM and Rad3-related (ATR) promote DNA damage response and stimulate the checkpoint protein kinases Chk1/2, that can influence cell cycle arrest. CDK1 and other important proteins related to cell cycle checkpoints (e.g. CDC25C and CDC25A), and DNA damage, were found to be the critical proteins inside this cluster. Oxidative stress often induces cell cycle arrest (Klein and Ackerman, 2003; Pyo *et al.*, 2013), in part through the degradation of the CDC25C protein through a Chk1 protein kinase-dependent pathway (Savitsky and Finkel, 2002).

Cell cycle arrest associated with complex mixtures of PAHs, metals, and other organic compounds upon exposure to coal mining residues has been observed *in vitro* (Tucker and Ong, 1985) and *in vivo* (Espitia-Perez *et al.*, 2018). More recently, exposure to benzo[a]pyrene (also present in the cluster) has been reported to induce cell cycle arrest and apoptosis in human choriocarcinoma cancer cells through the generation of ROS (Kim *et al.*, 2017).

### Systemic effects of IEs and PAHs in the cell cycle

As shown in Table 3, the GO analysis of clusters 9 and 12 revealed 14 main process annotations associated with the cell cycle and particularly Cr and benzo[a]pyrene. The main biological processes found in these clusters included the following: i) regulation of mitotic cell cycle, ii) cell cycle checkpoint and iii) the interphase of mitotic cell cycle. Several reports have demonstrated that more-than-additive mortality is common for IE/PAH mixtures. The PAH toxicity in individual aspects suggests that they modify the accumulation of IEs and improve element-derived reactive ROS. Redox-active elements (e.g., Cu and Ni) are also capable of enhancing the redox cycling of PAHs (Gauthier *et al.*, 2015). Several reports have implicated IEs as modifiers of P450 function and regulation, which implies that such elements could alter P450-mediated PAH mutagenicity and carcinogenicity (Peng *et al.*, 2015). Cr is typically used in coal mining processes (Pandey *et al.*, 2014) and is particularly associated with the fine fractions of PM (Kothai *et al.*, 2009). The genotoxic effects of Cr are predominantly the formation of oxidative adducts and apurinic/apyrimidinic lesions, eventually resulting in DNA breakage (Vasylkiv *et al.*, 2010). Additionally, Cr(VI) has been shown to be aneugenic, as revealed by both chromosome assays and centromere-positive micronuclei assays (Wise and Wise, 2010). However, the combined toxicity of Cr and benzo[a]pyrene has rarely been studied.

Interestingly, *in vitro* cell cycle analysis has demonstrated that mixtures of benzo[a]pyrene and metals reduce the cell population in the G1 phase and increase cell arrest or accumulation in the G2/M phase (Muthusamy *et al.*, 2018). Once more, the mechanisms suggested include oxidative stress (Fischer *et al.*, 2005), DNA repair alteration (Tran *et al.*, 2002), and suppressor protein TP53 inhibition (Chiang and Tsou, 2009). Particularly, *in vitro* exposure to a combination of benzo[a]pyrene with As, Cr and Pb increases the ROS-mediated oxidative stress in HepG2 cells (Muthusamy *et al.*, 2018). In this regard, within the same module, proteins regulated by oxidative stress and DNA damage inside the cell were also identified as bottlenecks (AKT1, JUN, and CREBBP) together with benzo[a]pyrene. Other trace species found in our IE analysis, such as SiO_2_, have also been found to cause DNA damage, oxidative stress, cell cycle arrest at the G2/M checkpoint and apoptosis synergistically in co-exposure with benzo[a]pyrene (Asweto *et al.*, 2017).

### Systemic effects of IEs in the cell cycle

This cluster (composed of clusters 2 and 4) addresses a particular area of interest in relation to whether metal ions and IEs interfere with other cellular responses to DNA damage, such as cell cycle progression and control. In clusters 2 and 4, AKT1, JUN and CREBBP and the TP53, CCNB1, CCNA2, CDK6, CDK2, CDK1, ATM, ATR, and CDK7 proteins were found to be bottlenecks together with Cr and S. The biological processes linked to this and its respective proteins are presented in Table 4.

Among all the chemical species present in coal mining environments, IEs, in particular, are capable of causing the most oxidative damage through the generation of ROS (Valko *et al.*, 2006). IEs can enter the body through inhalation or consumption of contaminated meals and then accumulate in the bloodstream (Schweinsberg and Von Karsa, 1990). These elements are deposited in tissues by various mechanisms (Bridges and Zalups, 2005) and may cause DNA damage. In this cluster, together with proteins regulated by oxidative stress and DNA damage, we also found proteins such as cyclins and cyclin-dependent kinases that have been reported to be down-regulated in response to ROS and are implicated in the induction of cell cycle arrest as one of the immediate defense mechanisms against genotoxic damage from oxidative stress (Burch and Heintz, 2005). Particularly, CCNB1 seems to be depleted in response to oxidative stress, causing the regulation of G2/M transit via the Chk1-Cdc2 DNA damage checkpoint pathway (Pyo *et al.*, 2013). Conversely, because altered cell cycle progression and/or cell cycle control and DNA repair inhibition have been observed under low, non-cytotoxic concentrations of metal compounds, some authors have suggested that inhibition could also be a result of the ability of metal ions to modify zinc finger proteins involved in cell cycle control and DNA repair (Hartwig *et al.*, 2002). Interestingly, some authors have reported the suppression of TP53-mediated cell cycle arrest in human breast cancer cells MCF7, as a response to DNA damage caused by Cd(II) (Méplan *et al.*, 1999). Other IEs involved in the modification of zinc finger proteins include Ni and Co (Hartwig and Schwerdtle, 2002). However, no similar implications have been reported for Cr and S.

As discussed in the previous section, Cr(VI) has been demonstrated to be consistently mutagenic in bacterial and mammalian model systems, and its carcinogenic activity is thought to be due to the induction of DNA damage generated by reactive intermediates, eventually resulting in DNA breakage (Vasylkiv *et al.*, 2010). Free radicals from SO_2_ metabolism, such as SO_3_^._^, SO_4_^._^, SO_5_^._^ may also induce DNA strand breaks (Meng *et al.*, 2005), and recent studies have confirmed that SO_2_ derivatives (bisulfite and sulfite) cause mitotic delay in cultured human blood lymphocytes in a dose-dependent manner (Uren *et al.*, 2014).

### Effect of El Cerrejón and Guacamaya coal exposure on alkaline and FPG high-throughput Comet assay

The results of the alkaline comet assay showed the presence of primary lesions (% DNA tail increase) in V79 cells exposed to ECCS (bituminous coal from El Cerrejón mine) and LGCS (sub-bituminous coal from La Guacamaya mine) for 24 h. Additionally, the results of the modified comet assay show that the cultures exposed to ECCS maintain the same levels of % tail DNA, wheras the cultures exposed to LGCS showed an increase in % tail DNA, when compared to the no-enzyme groups. These results could indicate oxidative damage. Previous studies on coal and its products demonstrated resulting DNA damage and oxidative stress induced by the presence of IE and PAH (Valko *et al.*, 2006; da Silva, 2016). Such results may also be due to compounds identified in the current study, in which we report various levels of inorganic elements (heavy metals) in the bituminous coal from ECCS and sub-bituminous coal from LGCS and high levels of chromium in the coal from LGCS. It was known that some IEs (heavy metals) could generate oxidative damage by generating ROS (Valko *et al.*, 2006). Multiple cellular processes including cell cycle checkpoint activation and DNA repair are typically initiated in response to such DNA damage (Dasika *et al.*, 1999; Lima *et al.*, 2016).

## Conclusions

**Figure 11:**
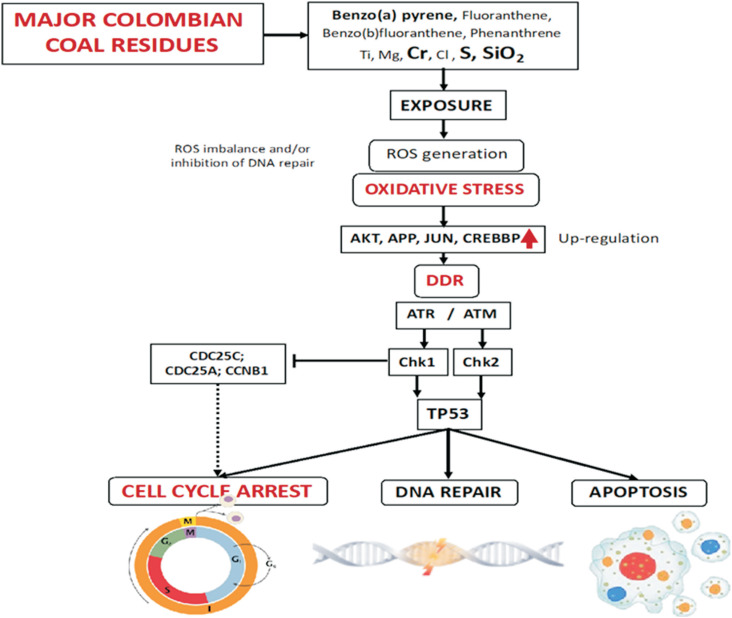
Molecular model illustrating how major coal residues potentially affect cell cycle progression: Exposure to major coal residues, such as benzo[a]pyrene, fluoranthene, benzo[b]fluoranthene, phenanthrene, Ti, Mg, Cr, Cl, S, and SiO_2_, can generate ROS via several pathways (e.g., Fenton-like reactions). The ROS imbalance and/or inhibition of the DNA repair process can lead to oxidative stress and the upregulation of several proteins associated with the oxidative response (AKT, APP, JUN and CREBBP) which are also involved in the control of the cell cycle. DNA and protein damage caused by the oxidative damage triggers DNA damage response mechanisms (DDR), including the protein kinase cascades ATM-Chk2/ATR-Chk1, which may result in cell cycle arrest. Oxidative stress can also induce cell cycle arrest through the degradation of CDC25C via the Chk1 protein kinase-dependent pathway. ATR phosphorylates and activates Chk1, which in turn, phosphorylates and inhibits Cdc25 phosphatases. Cdc25 inhibition ends up causing cell cycle arrest. Cdc25A phosphorylation by Chk1 triggers its degradation in a ubiquitin/proteasome-dependent manner. Both kinases phosphorylate TP53. In response to DNA damage, the activation of TP53 activates the expression of numerous genes involved in cell cycle arrest, DNA repair, apoptosis, and many other processes.

Using a systems chemo-biology approach, we examined how some of the major chemical constituents of coal dust and PM derived from coal mining activities interact with specific biological processes relation to the cell cycle. The main proteins and compounds present in the network were taken into account to construct a molecular model characterizing the effects of major coal residues on the cell cycle (Figure 11). The analysis performed in the present study suggests that coal residue MIOs (SiO_2_), IEs (Ti, Mg, Cr, Cl and S) and PAHs (benzo[a]pyrene, fluoranthene, benzo[b]fluoranthene and phenanthrene) can generate ROS. The resultant oxidative stress can induce cell cycle arrest through the upregulation of proteins such as AKT, APP, JUN and CREBBP, leading to DNA damage response activation by ATM/ATR and Chk1/Chk2 or by CDC25C or CCNB1 degradation. The model also suggested that protein p53 could be activated by Chk1/Chk2 and induce cell cycle arrest, senescence or apoptosis.

## Supplementary Material:

The following online material is available for this article:

Figure S1 Coal sample collection sites in Colombia.

Figure S2 Main CPI-PPI network generated by the Cytoscape 3.4.0 program.

Table S1 Major inorganic oxide components in coal ashes (%wt) as identified by XRF.

Table S2 IEs concentrations in coal samples as revealed by the PIXE assay (mean ± standard deviation).

Table S3 Polycyclic aromatic hydrocarbon concentrations per sample (mean ± standard deviation) as revealed by HPLC/UV/Vis.

Table S4 Proteins involved in hub-bottlenecks (HBs) and their function.
