# Supplementary material for: Systems chemo-biology analysis of DNA damage response and cell cycle effects induced by coal exposure
Source: Genet Mol Biol. 2020 Jun 26;43(3):e20190134. doi: 10.1590/1678-4685-GMB-2019-0134 (PMC7315349; doi:10.1590/1678-4685-GMB-2019-0134)

Genetics and Molecular Biology 43, 3 (2020)

Supplementary Material to "Systems chemo-biology analysis of DNA damage response and cell cycle effects induced by coal exposure"

Figure S1 - Coal sample collection sites in Colombia: 'El Cerrejón' (La Guajira, Colombia) and 'Guacamaya' (Puerto Libertador, Córdoba, Colombia) coal mines

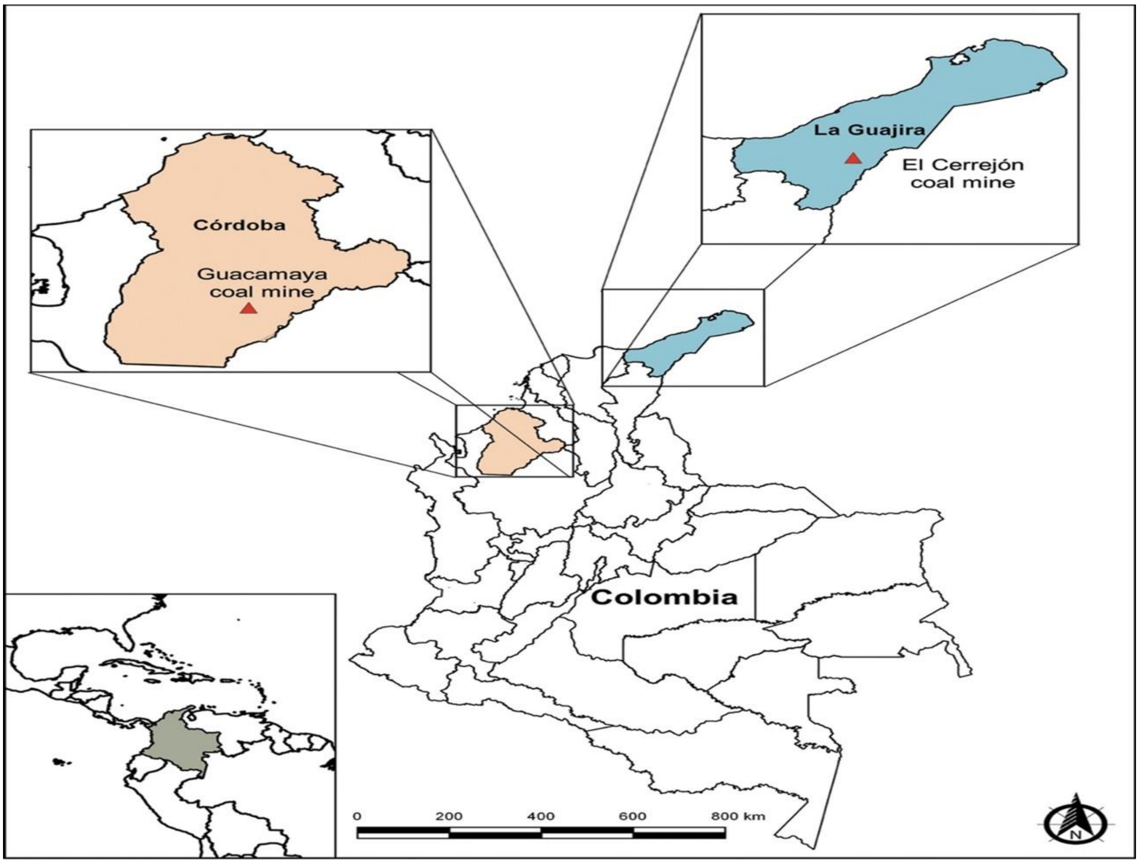

Supplement: Supplementary file 1 [file 1415-4757-GMB-43-3-e20190134-suppl1.pdf]
