# Supplementary material for: Systems chemo-biology analysis of DNA damage response and cell cycle effects induced by coal exposure
Source: Genet Mol Biol. 2020 Jun 26;43(3):e20190134. doi: 10.1590/1678-4685-GMB-2019-0134 (PMC7315349; doi:10.1590/1678-4685-GMB-2019-0134)
Supplement: Supplementary file 2 [file 1415-4757-GMB-43-3-e20190134-suppl3.pdf]

Genetics and Molecular Biology 43, 3 (2020)

Supplementary Material to "Systems chemo-biology analysis of DNA damage response and cell cycle effects induced by coal exposure"

Table S1 - Major inorganic oxide components in coal ashes (%wt) as identified by XRF

Elements	Guacamaya (%)	El Cerrejon (%)
**SiO_2**	13.01	62.21
**TiO_2**	0.33	0.92
**Al_2O_3**	7.52	19.62
**Fe_2O_3**	9.7	8.42
**MgO**	13.66	2.01
**CaO**	29.64	1.62
**Na_2O**	10.58	1.11
**K_2O**	0.44	2.17
**P_2O_5**	0.04	0.17
**SO_3**	15.08	1.75

%wt: percentage by weight
