# Supplementary material for: Systems chemo-biology analysis of DNA damage response and cell cycle effects induced by coal exposure
Source: Genet Mol Biol. 2020 Jun 26;43(3):e20190134. doi: 10.1590/1678-4685-GMB-2019-0134 (PMC7315349; doi:10.1590/1678-4685-GMB-2019-0134)
Supplement: Supplementary file 3 [file 1415-4757-GMB-43-3-e20190134-suppl5.pdf]

Supplementary Material to “Systems chemo-biology analysis of DNA damage response and cell cycle effects induced by coal exposure”

Table S3 - Polycyclic aromatic hydrocarbon concentrations per sample (mean ± standard deviation) as revealed by HPLC/UV/Vis

PAH	Guacamaya ($\mu g.kg^{-1}$)	El Cerrejón ($\mu g.kg^{-1}$)
**Naphthalene**	465 ± 16.34	864.1 ± 90.88
**Acenaphthene**	2.4 ± 0.03	3.8 ± 0.89
**Phenanthrene**	386.6 ± 1.46	846.2 ± 47.75
**Anthracene**	309.9 ± 13.37	1285.1 ± 110.57
**Fluoranthene**	1159.9 ± 59.17	1437.5 ± 245.52
**Benzo[a]anthracene**	676.5 ± 38.95	5045.4 ± 413.45
**Benzo[g,h,i]pyrene**	0.91 ± 7.60	1.3 ± 3.56
**Benzo[b]fluoranthene**	0.06 ± 0.02	0.6 ± 0.04
**Dibenzo[a, h]anthracene**	13.2 ± 0.001	28.9 ± 3.85
**Indeno[1,2,3-cd]pyrene**	9.5 ± 0.006	12.3 ± 0.005
**Benzo[k]fluoranthene**	0.9 ± 0.06	30.8 ± 1.10
