# Supplementary material for: Systems chemo-biology analysis of DNA damage response and cell cycle effects induced by coal exposure
Source: Genet Mol Biol. 2020 Jun 26;43(3):e20190134. doi: 10.1590/1678-4685-GMB-2019-0134 (PMC7315349; doi:10.1590/1678-4685-GMB-2019-0134)
Supplement: Supplementary file 4 [file 1415-4757-GMB-43-3-e20190134-suppl4.pdf]

Supplementary Material to "Systems chemo-biology analysis of DNA damage response and cell cycle effects induced by coal exposure"

Table S2 - Inorganic element concentrations in coal samples as revealed by PIXE assay (mean ± standard deviation)

Inorganic elements	Guacamaya (PPM)	El Cerrejón (PPM)
**Sodium (Na)**	**9212 ± 1050.73*****	3283 ± 285.97
**Magnesium (Mg)**	**4779.33 ± 332.27*****	2166.33 ± 167.63
**Aluminium (Al)**	1634.33 ± 75.22	**21311 ± 2110.55*****
**Silicon (Si)**	1341.67 ± 91.09	**37252.33 ± 3907.90*****
**Sulphur (S)**	4084 ± 312.64	**6807.67 ± 239.43*****
**Chlorine (Cl)**	52.94 ± 10.28	**243.77 ± 40.05****
**Potassium (K)**	137.33 ± 2.76	**1873.33 ± 180.79*****
**Calcium (Ca)**	**7838 ± 241.01*****	1675 ± 595.25
**Titanium (Ti)**	115.37 ± 14.79	**879.97 ± 241.27****
**Chromium (Cr)**	4.638 ± 3.591	20.98 ± 0.97
**Manganese (Mn)**	9.56 ± 1.38	**85.46 ± 18.21***
**Iron (Fe)**	3078.67 ± 76.63	**6113.33 ± 1399.06***
**Nickel (Ni)**	17.61 ± 21.44	241.77 ± 163.14
**Zinc (Zn)**	12.40 ± 1.71	**31.63 ± 6.89***
**Strontium (Sr)**	180.7 ± 8.54	ND

Unpaired T Test/ **Bold** for significantly higher values ***P < 0.001, **P < 001, *P < 0.05

ND = Not detected
