# Supplementary material for: Systems chemo-biology analysis of DNA damage response and cell cycle effects induced by coal exposure
Source: Genet Mol Biol. 2020 Jun 26;43(3):e20190134. doi: 10.1590/1678-4685-GMB-2019-0134 (PMC7315349; doi:10.1590/1678-4685-GMB-2019-0134)

Genetics and Molecular Biology 43, 3 (2020)

Supplementary Material to “Systems chemo-biology analysis of DNA damage response and cell cycle effects induced by coal exposure”

Figure S2 - Main CPI-PPI network generated by the Cytoscape 3.4.0 program. The final network shows 24 chemical elements detected in coal samples after chemical characterization (yellow), 2057 nodes (24 substances, 2,033 proteins), and 24,957 edges (connections).

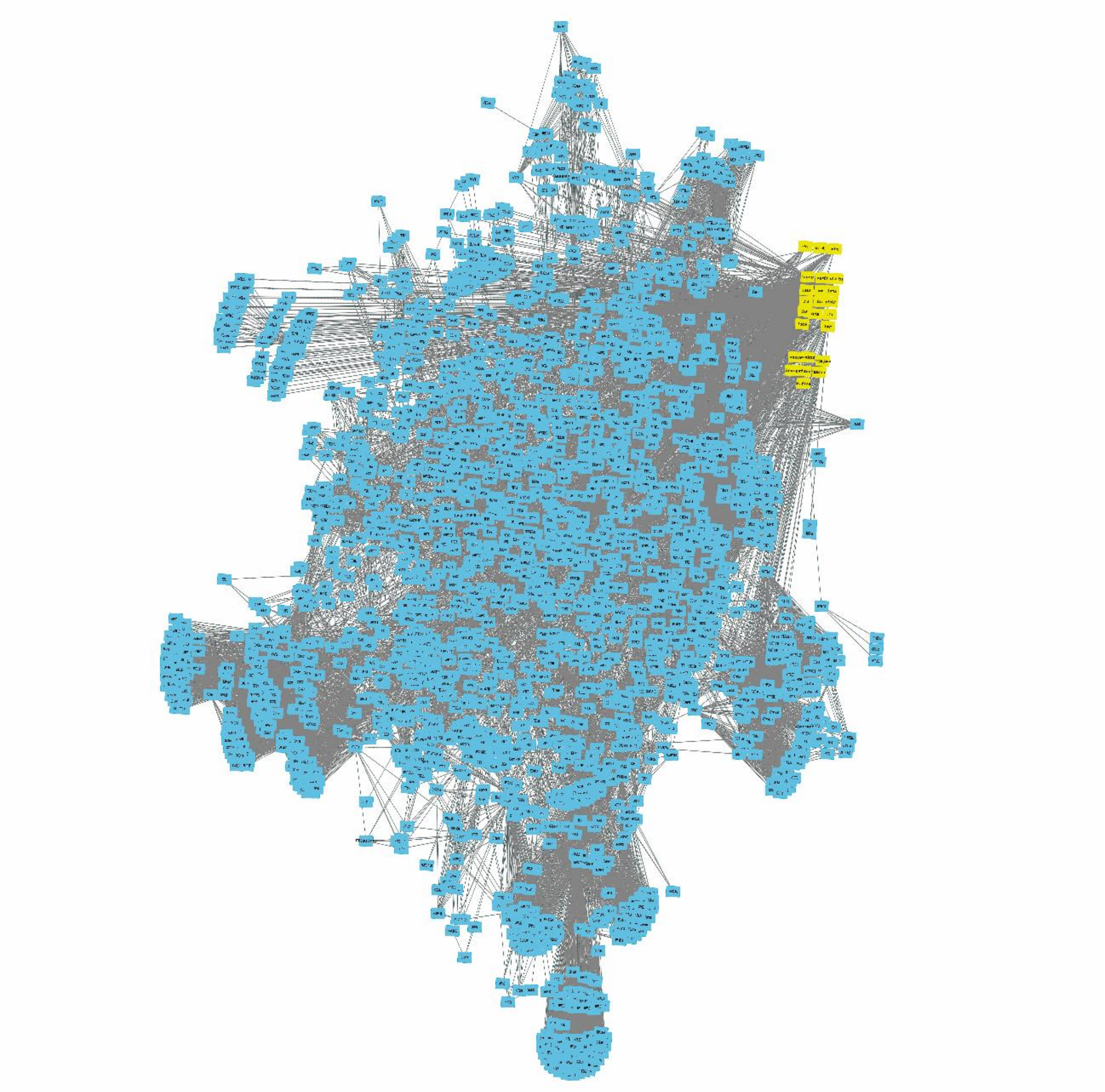

Supplement: Supplementary file 5 [file 1415-4757-GMB-43-3-e20190134-suppl2.pdf]
