# Supplementary material for: Systems chemo-biology analysis of DNA damage response and cell cycle effects induced by coal exposure
Source: Genet Mol Biol. 2020 Jun 26;43(3):e20190134. doi: 10.1590/1678-4685-GMB-2019-0134 (PMC7315349; doi:10.1590/1678-4685-GMB-2019-0134)
Supplement: Supplementary file 6 [file 1415-4757-GMB-43-3-e20190134-suppl6.pdf]

Supplementary Material to "Systems chemo-biology analysis of dna damage response and cell cycle effects induced by coal exposure"

Table S4. Proteins involved in hub-bottlenecks (HBs) and their function.

Protein name	Description [a]	Function [a]
UBC	Ubiquitin C	protease binding
UBA52	Ubiquitin A-52 Residue Ribosomal Protein Fusion Product 1	structural constituent of ribosome
RPS27A	Ribosomal Protein S27a	structural constituent of ribosome
HSP90AA1	Heat Shock Protein 90 Alpha Family Class A Member 1	identical protein binding
CAD	Carbamoyl-Phosphate Synthetase 2, Aspartate Transcarbamylase, And Dihydroorotase	protein binding and enzyme binding
SRC	SRC Proto-Oncogene, Non-Receptor Tyrosine Kinase	transferase activity, transferring phosphorus-containing groups and protein tyrosine kinase activity.
JUN	Jun Proto-Oncogene, AP-1 Transcription Factor Subunit	sequence-specific DNA binding
MAPK14	Mitogen-Activated Protein Kinase 14	transferase activity, transferring phosphorus-containing groups and protein tyrosine kinase activity
APP	Amyloid Beta Precursor Protein	protein binding and enzyme binding.
CREBBP	CREB Binding Protein	DNA-binding transcription factor activity and transcription factor binding
AKT1	AKT Serine/Threonine Kinase 1	protein binding and protein kinase activity

[a]The protein descriptions and functions were obtained from the GeneCards website [http://www.genecards.org].
